# Centrifugally Spreading Annular Erythema as a Dermatological Indicator of Metastatic Breast Carcinoma

**DOI:** 10.7759/cureus.51641

**Published:** 2024-01-04

**Authors:** Joohyung Youh, Yasuyuki Yamaguchi, Etsuo Hiraguchi

**Affiliations:** 1 Department of Dermatology, Hakodate Central General Hospital, Hakodate, JPN; 2 Department of Dermatology, Sapporo Dermatology Clinic, Sapporo, JPN; 3 Department of Surgery, Hakodate Central General Hospital, Hakodate, JPN

**Keywords:** cutaneous metastasis of breast cancer, erythema annulare centrifugum, annular erythema, skin metastasis, breast cancer

## Abstract

Breast cancer is the leading cause of skin metastasis in women with internal malignancies. This report highlights an atypical case of cutaneous metastasis of breast cancer (CMBC) in a 66-year-old woman. Starting four months before her dermatology consultation, the patient underwent a chemotherapy regimen comprising pertuzumab, trastuzumab, and vinorelbine for right breast cancer, right axillary lymph node enlargement, and bone metastases. After commencing chemotherapy, erythematous macules appeared around her right nipple. Subsequently, the cutaneous lesions developed into annular erythematous patches around her right nipple and began to coalesce and expand to the contralateral breast. A skin biopsy revealed dysplastic cells indicative of metastasis from invasive ductal carcinoma. In addition, lymphovascular tumor cell invasion was noted in the reticular dermis. Based on these clinical progressions and histopathologic findings, a diagnosis of CMBC was made, specifically considering the possibility of inflammatory breast cancer (IBC). The patient continued the same chemotherapy regimen for 17 cycles, which improved the skin lesions, but she succumbed to breast cancer two years later. This case emphasizes the importance of considering CMBC in breast cancer patients with expanding, treatment-resistant thoracic cutaneous lesions, especially in aggressive subtypes like IBC. The diverse presentations of CMBC require thorough histopathological evaluation.

## Introduction

Among the various internal malignancies in women, breast cancer shows the highest propensity for skin metastasis. Approximately 24% of women with metastatic breast cancer present with cutaneous manifestations [1-2]. The most frequent locations for cutaneous manifestations of breast cancer (CMBC) are the chest and abdominal areas, although they can also appear on the limbs and in the head and neck regions. Skin metastases originating from breast cancer may present in a variety of forms, including nodules, alopecia, telangiectasia, malignant melanoma-like metastases, carcinoma erysipelatoides, subungual metastases, carcinoma en cuirasse, zosteriform metastases, metastases to the eyelids, and Paget-like metastases [1]. The presentation of annular erythema as a cutaneous symptom of breast cancer is infrequently reported and might be associated with the lymphovascular invasion of the dermis by tumor cells. This type of CMBC is often related to inflammatory breast cancer (IBC), which is commonly characterized by breast enlargement accompanied by enlarging, diffuse erythema and a peau d'orange appearance [3-4]. In our report, we describe a unique clinical case of CMBC, characterized by rapidly, centrifugally spreading annular erythema.

## Case presentation

**Figure 1:**
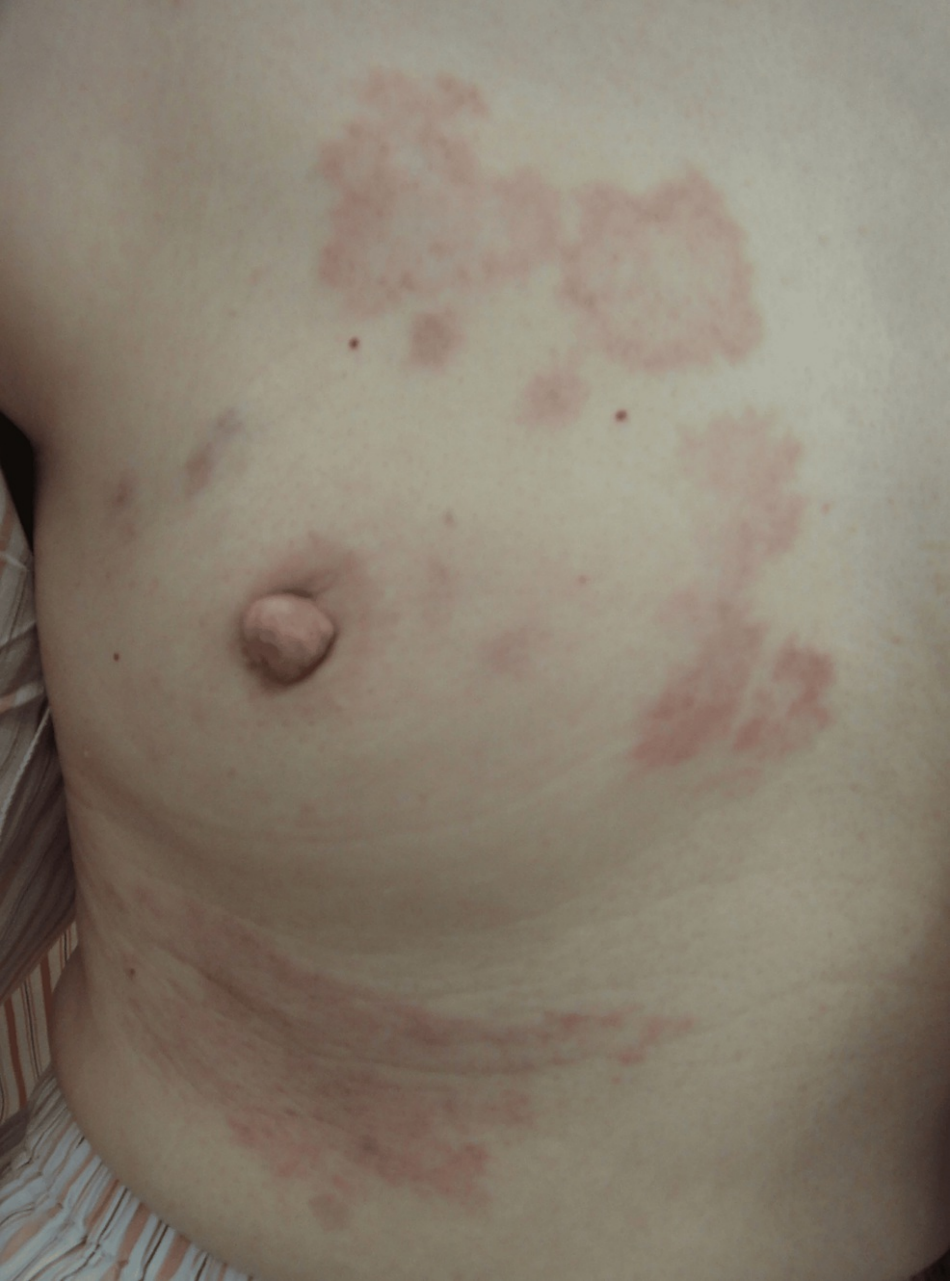
Clinical findings At the initial visit: annularly distributed, irregularly shaped erythematous patches and plaques around the nipple of the right breast, with noticeable partial central clearing

A 66-year-old woman presented with cutaneous lesions surrounding her right nipple. Upon physical examination, multiple non-palpable erythematous patches were observed. These were irregularly shaped and distributed in an annular pattern around the nipple of the right breast (Figure 1). The patient did not report any itching or burning sensations.

One year prior to her dermatology consultation, the patient noticed a subcutaneous mass on her right breast. Six months later, she visited the department of surgery at our hospital and was diagnosed with right breast cancer. Two weeks after the breast cancer diagnosis, multiple bone metastases, including in the right spine (Th10, L4) and ribs (R6-7), as well as right axillary lymph node enlargement, were detected in the imaging studies. Consequently, the department of surgery initiated a chemotherapy regimen comprising pertuzumab, trastuzumab, and vinorelbine, which began four months prior to her first visit to the dermatology department. She had no previous history of chemotherapy. Following the initiation of this chemotherapy regimen, cutaneous lesions appeared on her right breast.

**Figure 2:**
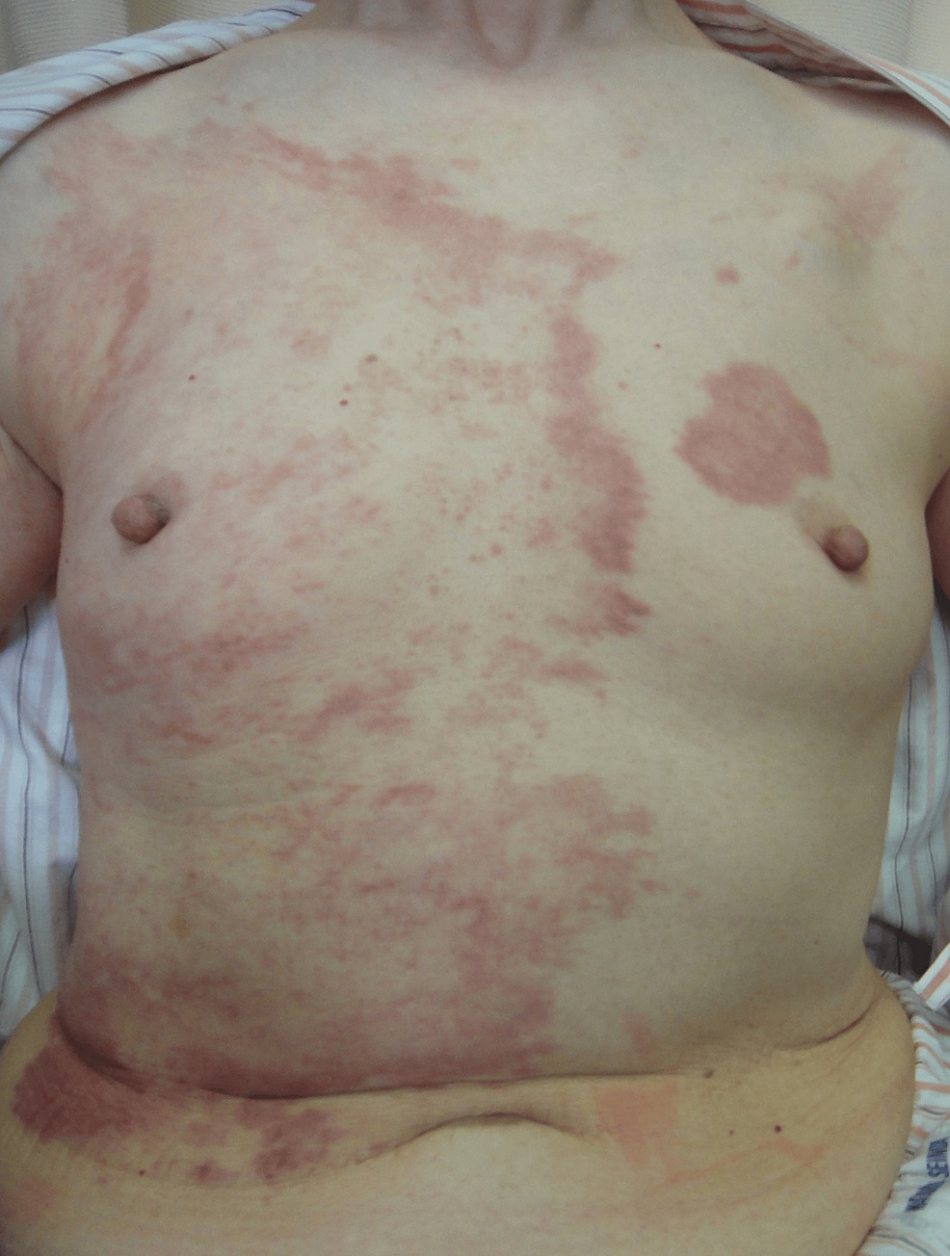
Clinical findings Ten months post-initial visit: expansion of the erythematous patches and plaques on the right breast, extending to the contralateral side

Initially, we commenced therapy with very potent topical corticosteroids, considering the possibility of a cutaneous adverse drug reaction (CADR) due to chemotherapy. However, despite a limited response to the topical corticosteroids, the lesions continued to expand and showed evidence of spreading to the contralateral side. The erythematous macules and patches on the left breast began to coalesce (Figure 2).

Because partial central clearing of the cutaneous lesions was noted in some areas of the rashes, we conducted a potassium hydroxide (KOH) test; however, it yielded negative results.

**Figure 3:**
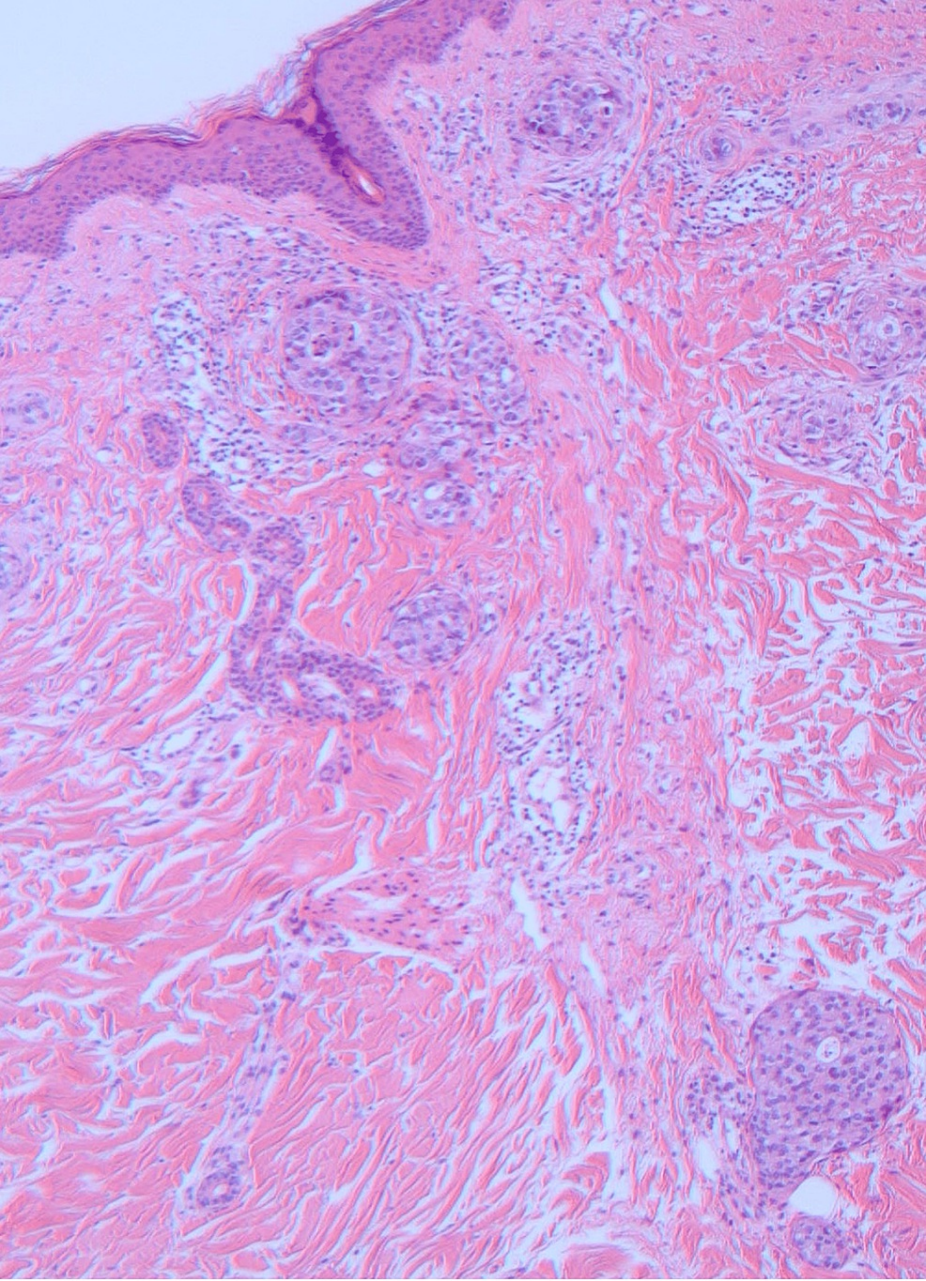
Histological findings Dysplastic cell infiltration with a high nuclear-cytoplasmic ratio in the superficial dermis and visible ductal formation was noted (hematoxylin-eosin stain, ×40).

**Figure 4:**
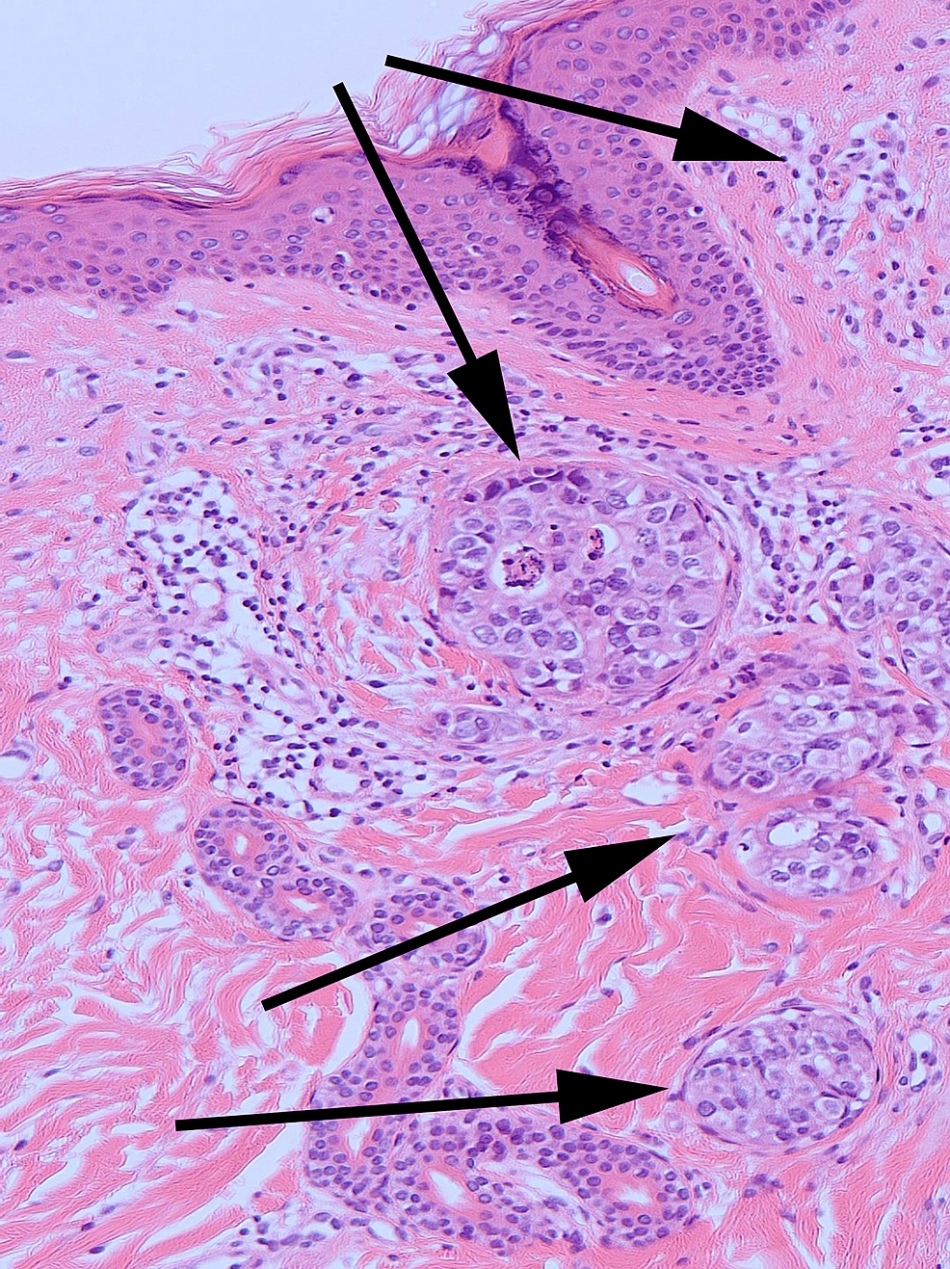
Histological findings Dysplastic cell infiltration with a high nuclear-cytoplasmic ratio in the superficial dermis and visible ductal formation was noted. The lymphovascular invasion of tumor cells, indicated by a black arrow, suggests inflammatory breast carcinoma (hematoxylin-eosin stain, ×100).

A skin biopsy of the newly appeared annular lesion on the left breast revealed dysplastic cell infiltration, characterized by a high nuclear-cytoplasmic ratio, in the superficial dermis (Figures 3 and 4). These observations were consistent with metastasis from invasive ductal carcinoma [4]. Furthermore, lymphovascular invasions of tumor cells, suggesting inflammatory breast carcinoma, were noted in the reticular dermis (Figure 4).

In addition, there was an overexpression of the human epidermal growth factor receptor 2 (HER2) and a notable increase in the Ki-67 proliferation index, which was recorded at 49.7%. Based on the clinical progression and histopathological characteristics, a diagnosis of CMBC was confirmed. 

After the CMBC diagnosis, the patient's chemotherapy was continued in the department of surgery. The same chemotherapy regimen, consisting of pertuzumab, trastuzumab, and vinorelbine, was administered for 17 cycles. This treatment led to a gradual reduction in the skin lesions. The erythematous plaques resolved six months after the initial consultation. Although the patient ultimately succumbed to the progression of breast cancer two years later, the cutaneous lesions did not recur.

## Discussion

Initially, based on the clinical progression, the likelihood of a CADR was considered. Given the diverse manifestations of CADR, it is essential to consider this possibility when new cutaneous lesions appear following the initiation of new medications or chemotherapeutic agents. However, no significant findings supported the diagnosis of CADR.

Subsequently, the observation of a central clearing pattern in some chest lesions, a characteristic feature of tinea corporis (a fungal infection), led to conducting a KOH test. The negative result ruled out a fungal infection.

Ultimately, the histopathologic findings, which included infiltration of dysplastic cells with a high nuclear-cytoplasmic ratio and visible ductal formation, as well as lymphovascular tumor cell invasions in the reticular dermis, pointed to a diagnosis of CMBC. Specifically, these findings suggested the possibility of IBC.

The thorax is the most common site for CMBC, accounting for 57% of cases, followed by the abdomen, axilla, and neck [2]. CMBC can mimic several conditions, including nodules and cutaneous inflammation seen in benign skin diseases, such as zosteriform metastasis and carcinoma erysipeloides [2]. Our review of the PubMed database from 1987 to 2022 revealed only six cases of CMBC presenting as annular erythema [3,5]. These lesions primarily appeared on the chest wall and spread to adjacent areas, with one case on the back [5]. In De Giorgi et al.'s review of 295 cases, nodular carcinoma was found in 46.8%, while erythema annulare centrifugum-like lesions, similar to our case, were observed in just one case [1]. This rarity of such cutaneous manifestations in breast cancer adds to the diagnostic challenge.

The annular erythema type of CMBC, mimicking erythema annulare centrifugum, often shows overexpression of HER2 and absence of hormone receptors. This subtype, marked by cancer cells invading dermal lymphatics but not the dermis, is typically linked to IBC. IBC, which makes up 2.5% of all breast cancer cases, is exceptionally aggressive, characterized by an increased risk of recurrence, more severe progression, and lower survival rates [6,7].

In approximately 75% of IBC cases, key histopathological signs, including scattered tumor emboli in skin biopsies or surgical specimens and dermal lymphatic invasion, are observed. While the presence of dermal lymphatic invasion is a diagnostic aid, it is not an absolute criterion. Notably, rapid changes in the skin over the affected breast, such as erythema and edema, occurring within three to six months, are indicative of IBC. These changes may spread across the entire breast and even to the opposite breast, impacting the mediastinum, upper extremities, and cervical region [7].

When encountering rapidly expanding cutaneous lesions on the chest, particularly in patients with a history of breast cancer, CMBC, specifically the cutaneous manifestation of IBC, should be considered. At such times, it is crucial for physicians to conduct a histopathological examination for further evaluation.

## Conclusions

In our report, we highlight a rare case of CMBC characterized by rapidly spreading centrifugal annular erythema. Physicians should include CMBC in the differential diagnosis for women presenting with persistent, treatment-resistant lesions on the thorax. Especially when these lesions show rapid progression and expansion, the possibility of the cutaneous manifestation of IBC should also be considered, given its often poor prognosis and the need for vigilant management.
